# Current Directions in the Auricular Vagus Nerve Stimulation II – An Engineering Perspective

**DOI:** 10.3389/fnins.2019.00772

**Published:** 2019-07-24

**Authors:** Eugenijus Kaniusas, Stefan Kampusch, Marc Tittgemeyer, Fivos Panetsos, Raquel Fernandez Gines, Michele Papa, Attila Kiss, Bruno Podesser, Antonino Mario Cassara, Emmeric Tanghe, Amine Mohammed Samoudi, Thomas Tarnaud, Wout Joseph, Vaidotas Marozas, Arunas Lukosevicius, Niko Ištuk, Sarah Lechner, Wlodzimierz Klonowski, Giedrius Varoneckas, Jozsef Constantin Széles, Antonio Šarolić

**Affiliations:** ^1^Institute of Electrodynamics, Microwave and Circuit Engineering, Vienna University of Technology, Vienna, Austria; ^2^SzeleSTIM GmbH, Vienna, Austria; ^3^Max Planck Institute for Metabolism Research, Cologne, Germany; ^4^Cologne Cluster of Excellence in Cellular Stress and Aging Associated Disease (CECAD), Cologne, Germany; ^5^Neurocomputing & Neurorobotics Research Group, Complutense University of Madrid, Madrid, Spain; ^6^Laboratory of Neuronal Networks, Department of Mental and Physical Health and Preventive Medicine, University of Campania “Luigi Vanvitelli”, Naples, Italy; ^7^Ludwig Boltzmann Cluster for Cardiovascular Research at Center for Biomedical Research, Medical University of Vienna, Vienna, Austria; ^8^Foundation for Research on Information Technologies in Society, Zurich, Switzerland; ^9^Department of Information Technology, Ghent University/IMEC, Ghent, Belgium; ^10^Biomedical Engineering Institute, Kaunas University of Technology, Kaunas, Lithuania; ^11^Faculty of Electrical Engineering, Mechanical Engineering and Naval Architecture, University of Split, Split, Croatia; ^12^Nalecz Institute of Biocybernetics and Biomedical Engineering, Polish Academy of Sciences, Warsaw, Poland; ^13^Sleep Medicine Centre, Klaipeda University Hospital, Klaipėda, Lithuania; ^14^Institute of Neuroscience, Lithuanian University of Health Sciences, Palanga, Lithuania; ^15^Department for Surgery, Medical University of Vienna, Vienna, Austria

**Keywords:** vagus nerve stimulation, auricular nerves, auricular transillumination, stimulation patterns, stimulation optimization, *in silico* modeling, personalized stimulation

## Abstract

Electrical stimulation of the auricular vagus nerve (aVNS) is an emerging electroceutical technology in the field of bioelectronic medicine with applications in therapy. Artificial modulation of the afferent vagus nerve – a powerful entrance to the brain – affects a large number of physiological processes implicating interactions between the brain and body. Engineering aspects of aVNS determine its efficiency in application. The relevant safety and regulatory issues need to be appropriately addressed. In particular, *in silico* modeling acts as a tool for aVNS optimization. The evolution of personalized electroceuticals using novel architectures of the closed-loop aVNS paradigms with biofeedback can be expected to optimally meet therapy needs. For the first time, two international workshops on aVNS have been held in Warsaw and Vienna in 2017 within the scope of EU COST Action “European network for innovative uses of EMFs in biomedical applications (BM1309).” Both workshops focused critically on the driving physiological mechanisms of aVNS, its experimental and clinical studies in animals and humans, *in silico* aVNS studies, technological advancements, and regulatory barriers. The results of the workshops are covered in two reviews, covering physiological and engineering aspects. The present review summarizes on engineering aspects – a discussion of physiological aspects is provided by our accompanying article (Kaniusas et al., 2019). Both reviews build a reasonable bridge from the rationale of aVNS as a therapeutic tool to current research lines, all of them being highly relevant for the promising aVNS technology to reach the patient.

## Introduction

Bioelectronic medicine progressively comes into focus as a non-pharmaceutical treatment option for various diseases. Here neuromodulation of the vagus nerve (VN) gained a special interest in recent years.

This review aims to summarize the contemporary views on the electrical stimulation of the auricular VN (aVNS) as a promising electroceutical therapy in humans. Catalysts were the first two international workshops on aVNS in Warsaw (February 16, 2017) and Vienna (October 26 and 27, 2017) within the scope of EU COST Action “European network for innovative uses of EMFs in biomedical applications (BM1309).” In particular, the present review summarizes and discusses technical issues, modeling concepts, regulatory and safety requirements, and novel architectures of open and closed-loop aVNS paradigms. A focussed review on the physiological role of VN including a biology-driven rationale for aVNS is provided in our accompanying article (Kaniusas et al., 2019).

We start with a short introduction on biophysical principles underlying aVNS and continue with technological issues on aVNS and the associated challenges from an engineering point of view. Then we revise *in silico* modeling to optimize aVNS technology and closed-loop aVNS to personalize aVNS therapy. Future directions in aVNS are identified to complement this review.

In short, VN plays a crucial role in sensing and regulating bodily states while forming brain-body connections. The complex anatomic and physiologic structure of VN yields challenges in the engineering of effective aVNS devices. In particular, specific fibers of VN determine their particular signaling properties and projection sites, which require aVNS to be tuned to specific stimulation patterns. These should consider the degree of myelination of VN fibers – myelinated A and B fibers intermingling with non-myelinated C fibers, their associated excitability and direction of the information transfer.

**FIGURE 1:**
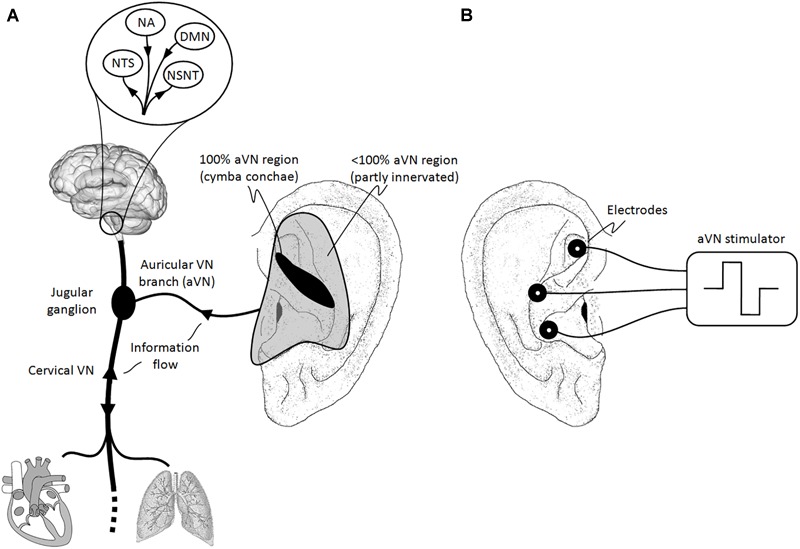
Natural sensory innervation of the auricle versus its artificial stimulation. **(A)** The vagus nerve (VN) connects the brain with most of the organs within the thorax and abdomen. Afferent auricular branches (aVN) leave the cervical VN at the level of the jugular ganglion just outside the cranium and innervate the rather central regions of the pinna of the outer ear (Peuker and Filler, 2002). **(B)** Electric stimulation of aVN endings with needle electrodes located within these central regions. NTS, nucleus of the solitary tract; NSNT, nucleus spinalis of the trigeminal nerve; NA, nucleus ambiguous; DMN, dorsal motor nucleus.

Most VN fibers (about 80%) are afferent sensory fibers carrying somatic and visceral information to the brainstem and thus providing a unique entrance to the brain (Berthoud and Neuhuber, 2001; Groves and Brown, 2005). As shown in Figure 1A, most afferent fibers of VN end in the nucleus of the solitary tract (NTS). The rest of VN fibers (about 20%) are efferent visceromotor fibers governing neurogenic, myogenic, and endocrine actions within projected organs.

From an engineering point of view, VN connects specific sensors and effectors in the periphery with the central nervous system. Mediated connections of VN include projections to brain regions mediating homeostatic signaling and multisensory integration and thus allowing a modulative access of VN to high-level brain functions (Berthoud and Neuhuber, 2001). Therefore, signals generated in VN have the potential to affect a broad range of basic brain functions and thus to affect the entire organism of the body in terms of its protection.

The external ear is an ideal place for a non-invasive or minimally invasive stimulation of VN. In fact, the auricular branch of VN surfaces as the auricular afferent VN (aVN) and thus forms a cutaneous receptive field in the pinna of the ear. Like VN, aVN is composed out of myelinated Aβ and Aδ fibers (Safi et al., 2016) and non-myelinated C fibers (Standring, 2016). The receptive field is susceptible to external stimuli in terms of peripheral nerve stimulation. In particular, aVN is available for an easy external access via electrical stimulation in terms of aVNS, which then connects directly and favorably the applied stimuli to the brainstem, as shown in Figure 1B. The auricle and especially its aVN endings might become a powerful direct gateway to the brain, offering the most affordable manipulation of the central nervous system.

Since aVNS projects directly to NTS (Figure 1A), both the autonomic and central nervous systems are modulated by aVNS. Consequently, since the autonomic nervous system, composed out of sympathetic and parasympathetic branches, governs systemic parameters of cardiovascular, respiratory, and immunological functions to stay within homeostatic limits and, on the other hand, aVNS modulates the parasympathetic aVN, aVNS effects on the body can be expected to be systemic.

Thus, aVNS is a peripheral, non-pharmacological, and minimally invasive neuromodulation technique. Due to systemic effects of aVNS – and, in general, of any vagus nerve stimulation (VNS) – many different biophysical mechanisms have been found to be modulated, as described in our accompanying article (Kaniusas et al., 2019). In short, aVNS alters signal processing in the central nervous system, activates reflex circuitries, and exploits brain plasticity and neural adaptation. The brain chemistry, nociceptive processing, inflammation, and autonomic function are modulated for different therapeutic purposes. Disease mitigating effects and sustainable therapeutic applications range from chronic pain diseases, neurodegenerative and metabolic ailments to inflammatory and cardiovascular diseases, including modulated psychometric functions.

## Electrical Stimulation of VN

### Invasive VNS Versus Non-invasive VNS

The targeted stimulation of the afferent VN, i.e., the translation of artificial electrical impulses into natural action potentials traveling into the brain, relays basically on four methods considering invasive VNS, non-invasive VNS, non-invasive aVNS, and minimally invasive aVNS:

(i) An invasive stimulation of VN can be performed via implanted cuff electrodes at the cervical level wrapped typically around the left cervical branch of VN (Mertens et al., 2018). The invasive VNS typically uses a bipolar cuff electrode (e.g., VNS Therapy, Cyberonics) with a bipolar stimulation pattern, either with a fixed non-adaptive stimulation (e.g., 30 s ON and 5 min OFF) or on-demand adaptive stimulation triggered by the patient. The method is approved for epilepsy and depression. Implantation risks and high costs are present, irreversibility of the electrode implant, as well as infection-associated morbidity. Unfortunately, implanted electrodes recruit not only the targeted afferent fibers but also the (visceral) efferent fibers of the mixed cervical VN branch (Howland, 2014). This unwanted stimulation of motor VN fibers leads to unfavorable multiple side effects such as cough, voice alteration (hoarseness), swallowing difficulties, or bradycardia, present in up to 30% of patients (Liporace et al., 2001). These side effects exclude application of strong and bilateral stimuli limiting their potential efficacy (Mercante et al., 2018). Side effects are incongruently reported either to increase during the time of stimulation (Liporace et al., 2001) or decrease (Beekwilder and Beems, 2010; Ben-Menachem et al., 2015).
(ii) The afferent branches of the cervical VN can also be non-invasively stimulated via surface skin electrodes of a hand-held device applied at the neck (Barbanti et al., 2015; Gaul et al., 2016; Silberstein et al., 2016). The transcutaneous stimulation uses two surface electrodes and is intermittently activated (e.g., GammaCore, electroCore, Inc.), e.g., for 6 stimulation sessions per day for in total 12 min, each session lasting 2 min. The method is approved for migraine headache and episodic cluster headache, as a preventive and/or acute treatment at onset of attacks. In order to stimulate VN relatively deep under the neck surface, the stimulation is performed using bipolar bursts of a relatively high frequency of 5 kHz for the burst duration of 1 ms, with a periodic bursting of 25 Hz (Gaul et al., 2016). However, relatively strong currents are still required to circumvent the skin barrier. Since the induced stimulation fields in the neck are diffuse, a co-stimulation of cervical non-vagal nerves and their local endings, as well as unintended recruitment of efferent fibers with the associated adverse effects can be expected. These side effects are prickling at stimulation site, neck pain, dizziness, headache, nasopharyngitis, and oropharyngeal pain (Gaul et al., 2016).
(iii) The receptive field of afferent aVN endings can be stimulated via non-invasive surface skin electrodes on the outer ear, known as transcutaneous aVNS (Ellrich, 2011; Straube et al., 2015). The transcutaneous aVNS uses two surface electrodes and is intermittently activated (e.g., NEMOS, Cerbomed GmbH), e.g., for 3–4 stimulation sessions per day for in total 4–5 h, each session lasting at least 1 h. The method is approved for epilepsy, depression, pain, and migraine. Advantageously, only afferent VN endings are stimulated, avoiding the aforementioned side effects of the invasive VNS. However, as a possible disadvantage, relatively large surface electrodes yield diffuse stimulation fields. Therefore, not only vagal but also other non-vagal nerves in the ear can be expected to be stimulated (Figure 1A), implications of which are still controversial (see Stimulation Regions). For instance, psychometric effects of the transcutaneous aVNS were independent on the precise stimulation location (Kothe, 2009), which highlights the diffuse regime of the transcutaneous aVNS. In addition, relatively strong currents and good electrode contacts are required for the current stimuli to circumvent the skin barrier of the ear and still stay suprathreshold in regions innervated by aVN. The stimulation is safe (Badran et al., 2018b), the remaining side effects are mostly minor – as related to invasive VNS – and may include headache, pain and skin irritation at the stimulation site, and dizziness (Mertens et al., 2018).
(iv) A percutaneous minimally invasive aVNS (Kampusch et al., 2013) can be performed with miniature needle electrodes penetrating the skin in the targeted outer ear regions innervated mainly by aVN (Figure 1). The percutaneous aVNS typically uses 2–3 needle electrodes and is also intermittently activated (e.g., AuriStim or P-Stim, Multisana GmbH). For instance, an automatic duty cycle of 3 h ON and 3 h OFF lasts for over 1 week, with a net stimulation time of 12 h per 24 h. Thus, the percutaneous aVNS shows the longest net stimulation time per day when compared with the invasive and transcutaneous aVNS. The percutaneous aVNS is approved for chronic cervical pain, chronic low back pain, migraine, acute postoperative pain, and pain due to peripheral arterial occlusive disease.

In contrast to the transcutaneous aVNS, the small size of needle electrodes and the resulting spatially focussed stimulating fields favor precise and specific stimulation of the local afferent aVN endings, which avoids diffuse stimulation. In addition, electrode contact impedance is lower and more reproducible, favoring an energy-efficient stimulation. Minor side effects of the percutaneous aVNS are local skin irritation (dermatitis), local bleeding, pain at the stimulation side, and dizziness. The clinical incidence of skin irritation and inadvertent bleeding can be reduced down to only 0.05% using a transillumination technique of the ear, which visualizes auricular vessels to avoid random placement of needles and thus reduces the potential of bleeding (Kampusch et al., 2016; Roberts et al., 2016). Although stimulation devices use needle electrodes and have to be worn over several days, more than 80% of patients are greatly satisfied with this treatment in terms of their subjective perception on life quality, with absent or only minor adverse effects (Kampusch et al., 2016).

A few indirect but rather seldom effects can also be triggered by aVNS due to afferent-efferent vagal reflexes with the vagal nucleus, NTS, as a potential intermediate stage. The Arnolds ear-cough reflex is the most dominant reflex, in which mechanical irritation/palpation of the auricular skin with embedded aVN may cause cough. There are also other reflexes as ear-gag reflex, ear-lacrimation reflex, ear-syncope reflex (known also as auriculo-cardiac reflex), and vaso-vagal reflex. These vegetative reflexes or reactions can occur with a delay of a few seconds (Tekdemir et al., 1998) and with the respective incidence up to a few percent in the general population (Tekdemir et al., 1998; Ellrich, 2011; Napadow et al., 2012). Syncope, tachycardia, bradycardia, paresthesia, vertigo, headache may also occur in response to aVNS. Contraindications for aVNS include immunocompromised patients (because of semi-permanent needles in the ear), hemophilia, psoriasis vulgaris at application site, the presence of a pacemaker or other active implantable devices (to avoid interference with aVNS), and vagal hypersensitivity.

In general, non-invasive and minimally invasive aVNS methods show fewer side effects than implantable VNS, which raises the potential number of patients who could benefit from aVNS. aVNS targets to modulate specific functions of the brain (Kaniusas et al., 2019) and thus to reach a maximum therapeutic effect while minimizing side effects. Non-invasive portable devices are relatively easy to apply and are cost effective as related to implantable devices (Morris et al., 2016). Favorably, motor VN fibers cannot be recruited in aVNS avoiding a lot of potential inadvertent effects, which is in clear contrast to the invasive VN stimulation. Interestingly, scarification and cauterization of the outer ear was practiced earlier as a “gross acupuncture” for healing purposes, as a forerunner for aVNS.

### Optimization of Stimulation Settings in aVNS

Basically, two optimization aims should be addressed by the stimulation set-up of aVNS:

(i) The stimulation of vagally innervated regions of the ear should be targeted when applying aVNS. On the other hand,
(ii) the electrical stimulation should optimally recruit aVN fibers close to the stimulation electrodes for a given stimulation waveform and strength.

#### Stimulation Regions

The first aim can be practically addressed via the anatomical map of the different auricular nerves (Figure 1A). aVNS is typically performed in the cymba and cavity of concha, crus of antihelix, and the inner tragus region of the ear, which were found to be at least partly innervated by aVN (Alvord and Farmer, 1998; Kandel et al., 2000; Peuker and Filler, 2002; He et al., 2012). While the diffuse stimulation of the transcutaneous aVNS does not allow a precise selection of stimulated regions, a spatially focussed stimulation is favored by the percutaneous aVNS with its miniature needles set in the anatomical regions innervated by aVN.

A special recognition is required here on the recent controversy on the true anatomical location of aVN and whether the stimulation effects of aVNS are due to the recruitment of aVN or other nerves. The literature on the definite innervation of the auricle is very sparse and is usually based on often cited findings in Peuker and Filler (2002). These findings unfortunately show some inconsistencies with respect to aVN innervation regions (Burger and Verkuil, 2018) and are based on only 14 ears of seven human cadavers (Peuker and Filler, 2002), which may not reflect anatomical variations of a wider population.

Auricular vagus nerve stimulation is usually performed at the tragus or (cavum, cymba) concha. However, some approaches cover larger areas of the auricle (Sator-Katzenschlager and Michalek-Sauberer, 2007) with the potential – and even with the targeted aim (Kovacic et al., 2017) – to stimulate concomitantly a few more auricular nerves in addition to aVN. The co-stimulated nerves are the great auricular nerve (with connections to the spinal cord) and/or the auriculotemporal nerve (connecting to the nucleus spinalis of the trigeminal nerve). For instance, tracing of the transcutaneous stimulation at the tragus in rats labeled mainly the dorsal horn of the cervical spinal cord and labeled only sparsely NTS, the termination site of aVN (Figure 1A), as reported recently in Mahadi et al. (2019).

It is also discussed if the tragus includes aVN endings or only non-aVN endings, such as the great auricular nerve and the auriculotemporal nerve (Badran et al., 2018a; Burger and Verkuil, 2018). A potential recruitment of these nerves would suggest that mechanisms may be involved for tragal stimulation beyond those anticipated for the sole aVN stimulation. Potentially, the afferent and concomitant stimulation of vagal and non-vagal endings synergize each other. Only the cymba concha was found so far to be solely innervated by aVN (Peuker and Filler, 2002) with the associated maximum activation of vagal projections in NTS during stimulation, as compared to tragus, cavum concha, or earlobe stimulation (Yakunina et al., 2016). However, the cymba concha offers some disadvantages in terms of complexity of electrical stimulation by requiring to insert and/or to hold an electrode against the concha, as opposed to having to clip onto the tragus. These uncertainties impede a proper interpretation of stimulation effects while an optimal target for the auricular stimulation is still under debate (Badran et al., 2018a; Burger and Verkuil, 2018).

**FIGURE 2:**
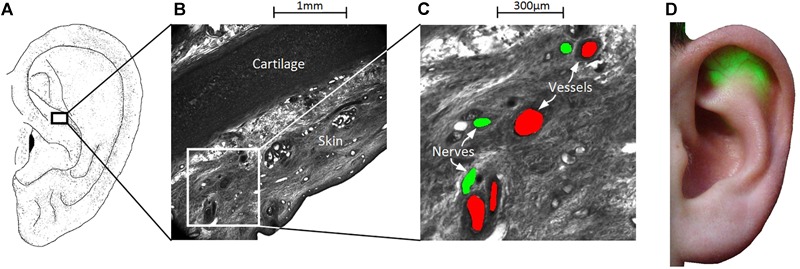
Wiring of vessels and nerves in the ear for the percutaneous aVNS. **(A–C)** High-resolution episcopic images of a volume biopsy in the cymba conchae of one male cadaver ear. Indicated blood vessels (in red) and nerves (green) reside apparently close to each other indicating their joint proliferation in the ear. **(D)** In order to find local auricular nerve branches, the outer ear is transilluminated to localize and visualize easily discernable auricular vessels which are less transparent than the surrounding tissue for green light. The visualized locations of vessels indicate the most likely regions of nerves, which serve for a personalized placement of stimulation needles.

Another important question here is how to find the individual aVN fibers in the ear and thus optimal regions for needles placement. In fact, aVN fibers and their bundles are too thin – in the submillimetre range (<100 μm), see Figure 2C – to be recognized visually by the unaided applicant’s eye. We have proposed (Kaniusas et al., 2011) to find these fibers based on the associated auricular blood vessels (Alvord and Farmer, 1998; Tilotta et al., 2008) since fibers and blood vessels are wired together, often alongside one another (Carmeliet and Tessier-Lavigne, 2005), even in the auricle (Razlighi et al., 2018). Figure 2B,C illustrate the joint proliferation of auricular vessels and nerves – based on episcopic images – with a separation distance of up to about 200 μm in this example.

A special transillumination method was designed to visualize auricular vessels to the applicant’s eye while making use of the different optical properties of blood vessels and the surrounding tissues (Kaniusas et al., 2011). In clinical practice, easily discernible vessels (<500 μm in auricle) indicate to the physician the most likely regions of local bundles of nerve fibers which are indistinguishable to the human eye. Figure 2D illustrates transilluminated ear with clearly visible auricular blood vessels.

Lastly, stimulation of the left or right aVN cannot be expected to yield different physiological effects since afferent information from both sides are centrally merged in the brainstem (Chen et al., 2015). This is in clear contrast to the invasive cervical VNS with dominant lateral effects, in which, for instance, the right side stimulation recruits predominantly the sinoatrial node (e.g., with the associated bradycardia) and the left side the atrioventricular node. However, simultaneous activation of the left and right aVN may potentially boost stimulation effects due to increased sensory input to the brainstem.

#### Stimulation Patterns

The second aim with the optimal recruitment of aVN fibers near electrodes is even less straightforward. The diffuse mode of the transcutaneous aVNS per-se does hardly allow a targeted optimisation of stimulation of particular aVN fibers, whereas local over-stimulation and under-stimulation of fibers are possible. This is in contrast to the percutaneous aVNS, in which stimulation needles can be precisely positioned relative to aVN fibers and thus the associated stimulation patterns may be optimized more easily; of course, provided that individual locations of aVN fibers are known.

Needles should reside close to the local aVN fibers or their endings (Bermejo et al., 2017) to guarantee their suprathreshold stimulation but not too close to avoid their cathodic block [characterized by the inhibited propagation of excitation (Kaniusas, 2019)]. Then the maximum recruitment efficiency can be attained for a given stimulation pattern (Tarnaud et al., 2018). In general, the thicker fibers are and the closer fibers are to the stimulation electrode, the less is the required excitation stimulus; in addition, myelinated fibers are excited more easily than non-myelinated fibers of the same thickness. Furthermore, more than one stimulation needle close to local aVN fibers can be expected to increase the net number of stimulated aVN fibers and thus to increase the net recruitment efficiency. The penetration depth of needles should be in the range of 1 mm, as suggested by the depth of auricular fibers under the skin (Bermejo et al., 2017; Razlighi et al., 2018).

Auricular vagus nerve stimulation of a percept-matched and subjectively comfortable intensity is preferred to reach therapeutic target (Busch et al., 2012; Napadow et al., 2012) which is a strong function of the stimulation pattern (Bald, 2010; Kaniusas, 2019). For instance, Deuchars et al. (2017) prefer intensities which are slightly below the subjective perception level. We hypothesize that a tingling sensation is necessary, in line with numerous studies (Kothe, 2009; Ellrich, 2011; Garcia et al., 2017; Sclocco et al., 2019). This is because the non-nociceptive aVNS should recruit myelinated Aβ fibers in the ear responsible for cutaneous mechanoreception and touch sensation, instead of myelinated Aδ fibers for cutaneous pain and temperature sensation. Advantageously, relatively thick Aβ fibers (with the diameter 7–10 μm) can be easier recruited than relatively thin Aδ fibers (2–5 μm).

In particular, the pain perception – accompanied typically by unpleasant, pricking, or burning sensations – should be avoided while stimulating (Ben-Menachem et al., 2015). Yuan and Silberstein (2015) report aVNS stimulation intensity between the patient’s detection threshold and the pain threshold in order to activate myelinated fibers. Ellrich and Lamp (2005) suggest that non-painful innocuous peripheral nerve stimulation preferentially activates Aβ fibers but not Aδ nociceptive fibers.

The selectivity in the stimulation of Aβ and Aδ fibers highly depends on the stimulation pattern, as supported by the experimental evidence (Dietrich et al., 2008). Here relatively high stimulation frequencies of 20–25 Hz are required for the peripheral electrical stimulation of the parasympathetic system, whereas low frequencies of 0.5–10 Hz are required for sympathetic system. High frequencies show narrow depolarizing half-period durations and thus are only able to recruit easily excitable thick nerve fibers (Kaniusas, 2019), such as myelinated Aβ fibers, which may indirectly activate the parasympathetic system. In contrast, wide depolarizing half-periods of low frequencies are required to recruit thin nerve fibers, such as myelinated Aδ or non-myelinated C fibers, activating usually the sympathetic system.

Different stimulation patterns are being used in aVNS. For instance, the percutaneous aVNS uses typically monophasic rectangular pulses every 1 s (1 Hz stimulation) with changing polarity and the pulse width of 1 ms, with the possibility to change settings (Kampusch et al., 2013). The pulse width usually determines the type of fibers to be excited. That is, short pulses recruit easily excitable thick fibers only while elongated pulses recruit both thick and thin fibers (Kaniusas, 2019). For the transcutaneous aVNS, authors in Badran et al. (2018b) show that different stimulation parameters yield different responses in the heart rate (systemic body parameter), whereas Polak et al. (2009) use vagus somatosensory evoked potentials to optimize parameters. It highlights the physiological and therapeutic relevance of the selected stimulation parameters.

The efficiency of stimulation can be increased with bursted stimulation (Martlé et al., 2014; Szabó et al., 2017); e.g., with bursts of short pulses every second (Reilly and Diamant, 2011; Kampusch et al., 2013; Kaniusas, 2019). A single or a few action impulses triggered at the sensory aVN endings in response to single electrical stimuli are less likely to influence systemic regulation or brain activity (e.g., the sympathovagal balance), rather than a rhythmic sequence of these impulses. This is because gradual natural sensory information is coded as the gradual temporal density of non-gradual impulses, likewise, coded as the instantaneous frequency of impulses. On the other hand, the brain with its very large number of neurons and its sophisticated processing is not likely to respond reasonably to a single or a few impulses but to a train of impulses.

Due to complex physiology of the body, continuous and intermittent stimulation, as well as strong and moderate stimulation, may even induce opposite physiological effects. For instance, synergistic actions of both sympathetic and parasympathetic systems were shown for continuous VNS (e.g., 10 Hz stimulation with 0.1 ms rectangular pulses), whereas antagonistic actions were demonstrated for intermittent VNS (e.g., 10 s ON period followed by 50 s OFF period) (Buchholz et al., 2014). Here, for instance, the continuous VNS was suggested to produce strong bradycardia and increased loading conditions of the heart, leading to compensatory sympathetic reflexes. In contrast, the intermittent VNS was, on average, not intense enough so that the parasympathetic VNS was still able to antagonize the sympathetic system.

Obviously, VNS or aVNS should be optimized with respect to the administered dose and duty cycle, whereas the ALARA principle (“as low as reasonably achievable”) applies for a given therapeutic indication. Favorably, VNS or aVNS should not be chronic in order to attain sustainable therapeutic effects. For instance, the activation of the anti-inflammatory response required only a brief VNS stimulus and lasted for more than 24 h (Olofsson et al., 2015). A sustained antinociceptive effect of aVNS was also observed in chronic low back pain for a 3 months follow-up after 6 weeks of treatment (Sator-Katzenschlager et al., 2004). In addition, the time instance of the stimulus with respect to inner body rhythms – such as heart beat or respiration – seems to be of high relevance. Ground-breaking experimental works in Brown and Eccles (1934) have shown the timing relevance in VNS. The influence of the timing between aVNS and respiration cycle was demonstrated in healthy subjects (Sclocco et al., 2019), as well as in chronic pelvic pain (Napadow et al., 2012), and migraine (Garcia et al., 2017), showing that aVNS delivered during exhalation was more efficient in brain, cardiovagal, and pain modulation than inspiration-gated stimulation.

While applying energy to the body via aVNS, electrochemical and metabolic stress factors have to be avoided (Kaniusas, 2019). The electrochemical stress is due to irreversible electrochemical reactions at the electrode/tissue boundary, potentially harming biological tissue, and is proportional to the absolute current level. This is in contrast to the metabolic stress as an integral stress, which is due to axonal loss and demyelination, and is proportional to the time integral of the stimulation current. In order to avoid irreversible electrochemical reactions, charge-balanced current stimulus is a necessary condition; however, strictly speaking, the charge balance is not a sufficient condition due to ongoing inert diffusion processes of reactants and products from/to the electrode/tissue boundary (Kaniusas, 2019).

## Regulatory Issues of aVNS Devices

Bringing a medical device, like an aVNS device, on the market comes along with many requirements since the medical device market is highly regulated. Requirements encompass research and engineering issues, clinical studies, regulatory and business issues. Even though these requirements vary for different types of stimulators, common regulatory requirements apply for all types of aVNS devices and thus deserve a short overview. Here we focus on EU guidelines and do not intend to cover global regulatory issues because of their diversity.

**FIGURE 3:**
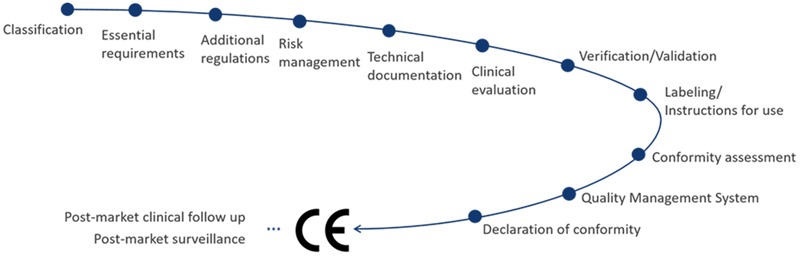
CE-related regulatory pathway of aVNS medical devices.

The medical device directive EU 93/42/EEC is the basis regulatory document for the required CE certification of aVNS devices in EU, for monitoring and reporting requirements as well as registration duties of manufacturer. A new regulatory framework, the Medical Device Regulation EU 2017/745 was released in 2017 and will only apply in spring 2020 after a transition period. In fact, CE certification is the trading passport in EU and European free trade association countries. The typical path for CE marking according to EU 93/42/EEC is illustrated in Figure 3.

Auricular vagus nerve stimulation device has to meet the essential requirements of the directive and fulfill the selected conformity assessment, which depends on the risk classification of aVNS device. The applicable risk classification specifies the quality management system that has to be implemented by manufacturer and the necessity of a notified body to control the conformity. In particular, a percutaneous aVNS device can be classified as “short term” (application for less than 30 days), “surgically invasive” (due to needle electrodes in the ear), and “active therapeutical” device (stimulation energy is applied), which implies that it is a Class IIa medical product. The following conformity assessment procedures are available for manufacturers for a Class IIa product: (i) verification of every device or a random sample of devices by the notified body, (ii) production quality assurance, (iii) product quality assurance, and/or (iv) a full quality assurance system.

The implementation of a risk management system is mandatory, accompanies the whole life cycle of any aVNS product, and serves as the input for development, design, and manufacturing of aVNS device. For instance, the risk management should guarantee that the exchange of energy with the human body is not hazardous, taking into account the nature, the density, and the site of the energy application. In addition, a usability engineering process needs to be established to assess the usability and possible use errors already during the development of aVNS, which is important for the final validation of aVNS device within the intended use or patients.

For state-of-the-art development and safety of aVNS device and its conformance with essential requirements, harmonized EU standards and guidelines shall be followed. Standards include EN ISO 14971 (for risk management), EN ISO 13485 (implementation of quality management system), EN 60601-1 including collateral standards (medical device basic safety and essential performance), EN 62366-1 (usability engineering), and EN 62304 (medical device software). Guidelines include MEDDEV 2.7/1 (for specification of a proper clinical evaluation) as well as MEDDEV 2.12-1 and MEDDEV 2.12/2 (market surveillance and vigilance). Many guidelines have been implemented within the new Medical Device Regulation and thus are now obligatory. Since aVNS device is body worn in home healthcare environments, additional requirements and limitations arise. Further requirements come from the required traceability and post-market surveillance that must be established to fulfill vigilance duties. National deviations in requirements have to be considered when marketing aVNS.

For the certification procedure and clinical safety, a rigorous clinical evaluation of aVNS device must be prepared and regularly revised, including clinical studies and/or a systematic literature review. Clinical evaluation can also be based on equivalent and predicate devices, proving technical, biological, and clinical equivalence with supporting data for all clinical claims made. However, showing the conformity by equivalence is getting less accepted. This can cause high costs and delayed market entrance due to the necessity of extensive clinical trials for each new derivative of aVNS device. A post-market clinical follow up plan is required. Side effects must be studied; for instance, the cardiac safety of the transcutaneous aVNS was investigated in Kreuzer et al. (2012), whereas most users were satisfied with the application and wear ability of the percutaneous aVNS (Kampusch et al., 2016).

Specific construction features of an aVNS device such as the implemented medical device software introduce further demands. Biocompatibility of the used materials, especially needle electrodes for the percutaneous aVNS, must also be proven. If aVNS device consists of several parts, e.g., separate stimulator and needle electrodes, these parts may need individual CE marking if supplied separately. In order to avoid harm to the patient (ICNIRP, 1998) – in addition to medical treatment effects – a risk analysis of electrode’s current densities higher than 2 mA/cm^2^ (EN 60601-2-10) has to be provided.

The notified body is needed for assessment of the technical documentation including the clinical evaluation, regular inspection of the quality management system, and, finally, for granting the CE certificate (for three years). Any minor or major findings during assessment or auditing have to be corrected within a specific time frame, followed by a report and a post-audit inspection. After a declaration of conformity is issued, the product for aVNS can be marketed within the EU after registration at each national level.

In summary, there is need for an appropriate regulatory management covering the whole life-cycle of any aVNS product to avoid failure in the conformity assessment and market entry. Highly faceted regulatory issues can delay time-to-market of an aVNS device and tremendously increase costs and workload for a medical device company. Thus, an early contact with a notified body already during development is recommended to prevent potential failures in the regulatory assessment.

## *In Silico* Numerical aVNS

In order to optimize aVNS – or any neurostimulation approach – numerical simulation of aVNS is a reasonable approach. The simulation requires a step-wise coupled electromagnetic and electrophysiological modeling. First, the distribution of the electric field in tissue is calculated in response to an applied electric stimulus; e.g., via current or voltage electrodes on the auricular skin (Figure 1B). The resulting distribution takes into account the particular anatomy of the ear and its tissue heterogeneity in terms of varying local electrical properties of the auricular tissue (its conductivity and permittivity); anisotropy of tissue properties is also considered. Second, the local electric fields, their gradients and dynamic – resulting along extracellular spaces or trajectories of auricular axons and their endings – are used for the neural simulation. That is, the induced fields are used for the investigation of the non-linear response of axonal membranes to the applied stimulus. The dynamics of the electric field is tightly connected with the temporal characteristics of the applied stimulus (e.g., with the pulse shape). For the neural simulation, the physiological distribution density of fiber types in the ear and their diameters as well as realistic fiber models are required. For instance, myelinated fibers can be approximated with the SENN model (Reilly and Diamant, 2011), the MRG model (McIntyre et al., 2002), or the Sweeney model (Sweeney et al., 1987). An integrated modeling with a feedback of the neural excitation to the excitatory extracellular electric potential is not reasonable since local action impulses with a typical swing in the membrane voltage by about 100 mV change only insignificantly the extracellular potential by 1–3 mV (Rattay, 1990). In short, electrical properties of tissues between electrodes and nerves and, on the other hand, properties of neural structures (fiber’s trajectories, type, and diameter) determine the physical stimulation depth and thresholds of aVNS for arbitrary electrode placement and stimulation waveform.

Effects of the electrode/tissue boundary have to be numerically accounted for. In particular, needle electrodes for the percutaneous aVNS act typically as polarizable electrodes, whereas surface electrodes for transcutaneous aVNS can act as non-polarizable electrodes. These different electrode/tissue interfaces can be numerically considered using pre-processed data as input into the electromagnetic modeling of aVNS.

**FIGURE 4:**
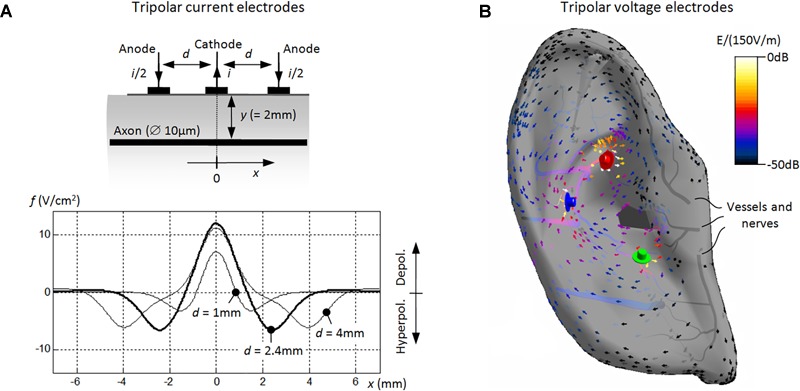
Numerical modeling of aVNS with three stimulation electrodes. **(A)** Basic model of the tripolar stimulation with surface current electrodes. A single cathode carries the current *i* (=1 mA) and the two surrounding anodes *i*/2 each. The unmyelinated axon lays in parallel to electrodes at the depth of 2 mm. Activating functions *f*(*x*) are shown along the axon’s coordinate *x*, showing the influence of the electrode separation *d*. With decreasing *d*, the depolarized segment of the axon narrows [i.e., Δx decreases for *f*(*x*) > 0] while the local depolarization strength decreases [i.e., *f*(*x*) decreases for *f*(*x*) > 0]. **(B)** Advanced model of the tripolar stimulation with needle voltage electrodes. The spatial distribution of the local electric field *E* (in dB related to 150 V/m) is shown within the outer ear with the electric potential 1 V for the red electrode, –1 V for the blue, and 0 V for the green. In fact, the gradient of the electric field [proportional to *f*(*x*)] determines the potential excitation of straight nerves along *x* aligned typically along auricular vessels (Figure 2).

Neurostimulation by an arbitrary number of active electrodes in the ear (single or multiple electrodes, see Figure 4B) can be numerically assessed. For instance, the concept of the activating function and their superposition can be favorably used here if continuous neuronal trajectories in the auricle are known (Rattay, 1986, 1999). It states that the activating function – proportional to the second derivative of the extracellular electric potential along the nerve trajectory – is positive for the local depolarization and is negative for the local hyperpolarization. The activating function predicts the site of spikes initiation for a given neuronal trajectory. Since the activating function of each single electrode is subjected to a strong decrease with *y*^3^ (Kaniusas, 2019), with *y* as the normal electrode distance to the considered axon (Figure 4A), a distant electrode with its *y* about three times of *y* of another near electrode can be neglected, if both electrodes carry the same stimulation current. Likewise, the distant electrode contributes only very little to the geometrical superposition result of the relevant activating functions. In contrast, discontinuous or bend neuronal trajectories are subjected to end-mode or bend-mode excitation, respectively (Kaniusas, 2019), which is particularly relevant in view of numerous aVN endings in the ear.

Different models for transcutaneous and percutaneous aVNS have been developed, which use multiple electrodes, consider spatiotemporal electric fields, the geometry and properties of embedded nerves (Kuhn et al., 2008; Samoudi et al., 2017, 2019; Kaniusas, 2019). These models allow numerical optimisation not only of an engineering aVNS solution (e.g., its electrode shape and the applied stimulation pattern) but also of the electrophysiological impact (e.g., the percentage of activated axons), and consequently of the potential therapeutic outcome of aVNS.

In particular, the required number of used stimulation electrodes for aVNS and the resulting spatial width of the current distribution within the ear can be numerically optimized. While monopolar aVNS offers the most diffuse stimulation with the lowest excitation threshold, bipolar and tripolar aVNS sharpen the stimulation focus but unfavorably increase the threshold (Reilly and Diamant, 2011; Kaniusas, 2019). In fact, the diffusivity should be potentially restricted to aVN regions in the ear (Figure 1A) to avoid stimulation of non-vagal nerves in the ear and to reach reproducible aVN-gated effects.

An optimal electrode position with respect to aVN fibers and the required penetration depth of needle electrodes for the percutaneous aVNS are other important optimisation parameters. The larger is the depth, the easier is the nerve recruitment, i.e., the lower are the activation thresholds of auricular axons and the larger is the percentage of activated axons. In particular, the closer is an electrode to the axon, the lower is its activation threshold. However, cathodic block with an inhibited propagation of action impulses can also arise for an axon residing too close. Therefore, the numerical simulation of aVNS indicates that the placement of needle electrodes should be ideally adapted to the individual vessel/nerve wiring of the ear. Simulations showed that a small distance of about 1 mm should be kept to vessels/nerves to avoid both subthreshold stimulation and cathodic block, as well as to avoid potential damage of vessel/nerves by the chronic electrode presence and to avoid local bleeding. Please recall that individual aVN fibers can be disclosed by the transillumination method (Figure 2D).

The temporal pattern and intensity of the applied stimulus can be optimized with respect to the required fiber recruitment and the selectivity of stimulation. In terms of selectivity, the simulation targets are Aβ fibers (responsible for mechanical sensing) but not Aδ fibers (for pain sensation) so that depolarising phases of the stimulus should be as short as possible to recruit easily excitable thick Aβ fibers but not hardly excitable thin Aδ fibers.

Cathodic stimulation requires lower thresholds than anodic stimulation given straight geometries of axons residing in a certain distance from the stimulation electrode. Monophasic pulses excite more easily than biphasic pulses, especially for short pulse durations <1 ms (Kaniusas, 2019). Therefore, cathodic monophasic thresholds are the lowest when individual nerve fibers are considered. However, if we are interested in maximizing the total recruitment volume in the ear enclosing multiple fibers at both anode and cathode, biphasic waveform seems to be in favor of monophasic waveform. Then numerous fibers below anode and cathode – disjoint fiber populations – would experience successive cathodic stimulation (with a low threshold) and anodic stimulation (with a high threshold). In particular, the largest recruitment volume results when the biphasic waveform includes an interphase interval lowering the stimulation threshold and thus increasing the recruitment volume even more (Reilly and Diamant, 2011). Of course, monophasic pulses should also be avoided to prevent the charge imbalance on the electrode/tissue boundary and thus to avoid irreversible electrochemical reactions (Kaniusas, 2019).

Another advantage of biphasic waveform is that it may enhance the total recruitment of fibers excited at their terminal endings and/or bend regions (Reilly and Diamant, 2011; Kaniusas, 2019) within the ear. Depending on the orientation of the bend region and/or the terminus of the neuron, monophasic stimulation leads either to depolarization or hyperpolarization of an exposed terminus or exposed bend region close to the electrode. In contrast, biphasic stimulation leads to depolarization of exposed termini and bend regions within a single cycle of biphasic waveform under each electrode. Therefore, biphasic stimulation may favorably enhance the number of excited disjoint fibers at each electrode due to the phase reversal.

Figure 4 shows *in silico* data of aVNS based on tripolar stimulation which has been shown to provide a more focussed, spatially selective stimulation of fibers than bipolar or monopolar stimulation (Kaniusas, 2019). Figure 4A illustrates increasing sharpness of the spatial stimulation area (in the range of 1 mm) with decreasing separation distance between the three stimulation electrodes based on the concept of the activating function. However, the improved focus in the tripolar stimulation arrives at the cost of a reduced stimulation efficiency and thus increased stimulation threshold (Figure 4A). This compromise is also valid for bipolar stimulation. Figure 4B illustrates maximum values of the local electric field and of its gradient close to the needle electrodes due to the electrical point effect (Kaniusas, 2019). The vector field flows in the direction from positive anode (red electrode) to negative cathode (blue electrode) within the outer ear, whereas local electric fields decrease along conducting blood vessels within the ear. While the resulting gradient of the local electric field along straight axons determines their local excitation, a constant electric field is sufficient to depolarize and potentially excite terminal endings and/or bend regions of auricular axons.

The design of electroceuticals such as aVNS devices can be assisted by computational models, i.e., functionalized anatomical models subjected to the discussed coupled electromagnetic and electrophysiological modeling. These neuro-functionalized models feature realistic nerve trajectories within detailed anatomical phantoms (Neufeld et al., 2016). Models facilitate the exploration and optimisation of various stimulation settings (e.g., electrode number, shape, and position, as well as stimulation waveforms) for an efficient recruitment of nerves while considering the complex heterogeneity of human tissues and specific anatomy; here extensive sensitivity analyses to settings changes can be realized. Such models have the potential to minimize the cost, time efforts, and the number of involved humans and animals in clinical and experimental trials, respectively, whereas safety and efficacy of electroceuticals is increased. Furthermore, functionalized phantoms can be personalized to arrive at planning and optimization of highly patient-specific treatment. For instance, such computational models can be generated with the Sim4Life platform (Neufeld et al., 2014) for life sciences investigations from Zurich MedTech AG (Zurich, Switzerland).

## Personalized aVNS

**FIGURE 5:**
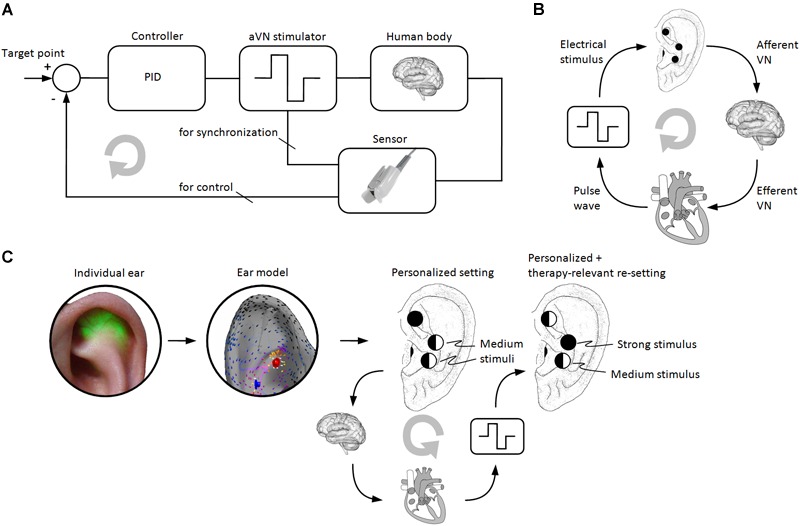
Personalized aVNS. **(A)** The closed-loop aVNS with the physiological biofeedback (e.g., magnitude of the pulse wave or HRV) which is used to control stimulation parameters of aVNS (e.g., the stimulus strength) in order to adhere to momentary therapeutic needs (e.g., optimal blood perfusion in legs). The biofeedback can also be used for a temporal synchronization of the applied stimuli with inner body rhythms (e.g., respiratory or cardiac cycle) to interfere constructively with the dynamics of the body. **(B)** aVNS of the afferent VN modulates activity of the efferent VN outflow to the heart, whereas the peripheral pulse wave arriving from the heart can be used as biofeedback to the stimulator of aVNS. **(C)** Personalized and optimized setting of stimulation needles and their stimulation patterns for the percutaneous aVNS based on an individualized ear model (Figure 4B) as derived from the transilluminated individual ear (Figure 2D). Therapy-relevant re-optimization of the stimulation patterns results when the closed-loop aVNS from the panels **(A,B)** operates. Here the level of the circle’s filling at each electrode position indicates the local stimulation strength which changes in the course of the closed-loop control.

Straightforward open-loop aVNS without any dynamic adjustment of stimulation parameters may be sufficient for treatment when the targeted neuromodulatory effects take a long time to establish, i.e., when there are large time constants involved. Here a periodic readjustment (e.g., weekly) of stimulation parameters of aVNS by physicians may be sufficient to account for these large time constants. However, for relatively acute treatments, a closed-loop aVNS with an instantaneous biofeedback – as shown in Figure 5 – may be more favorable where stimulation parameters are adapted based on the concurrently recorded physiological impact of aVNS. A real-time adaptation of the aVNS stimulus is reasonable to control excitation of aVN according to momentary therapeutic needs and the actual physiological state of the body.

Each individual patient can be expected to respond uniquely to aVNS therapy, e.g., findings in Frei and Osorio (2001) reveal variable effects of VNS on the heart rate (bradycardia or tachycardia) between patients but consistent within patients. Therefore, the commonly delivered open-loop aVNS may significantly limit the aVNS effectiveness unless a proper adaptivity of aVNS through biofeedback is established within a patient. In contrast to the open-loop aVNS, the closed-loop aVNS adapts rapidly to changing conditions and thus offers a personalized aVNS for a personalized disease control with increased therapeutic efficiency, raised quality of life, and reduced severity of side effects. For instance, the closed-loop deep-brain stimulation in rats showed a reduction in the seizure frequency by 90% versus only 17% in the open-loop stimulation (Salam et al., 2015); more examples on the closed-loop stimulation will follow. In addition, the closed-loop stimulation was shown to save energy by up to 42% in deep-brain stimulation (Grant and Lowery, 2012). A systematic overview of concurrent sensing and stimulation technologies in chronic closed-loop neuromodulation devices can be found in Stanslaski et al. (2012).

### Formation of Biofeedback

The easiest way to close the loop is to provide a simple on-demand activation of aVNS via subjective biofeedback or via a biomarker from the patient (e.g., activation via button of magnet stick in response to upcoming pain). However, besides missing objective physiological data, elderly and diseased patients cannot be expected to comply with this self-governed feature for a variety of reasons (Kampusch et al., 2016). In addition, subjective biofeedback is possible in specific diseases only (e.g., chronic back pain) which severity can be individually perceived by the patient.

Thus, an algorithmic-driven activation/adjustment of aVNS is necessary based on individual physiological biofeedback provided back to the stimulator (Figure 5) and using different control models (Romero-Ugalde et al., 2015, 2017). Recording and analysis of diverse biosignals in response to aVNS can close the loop and thus allow optimization and personalization of aVNS therapy. The particular choice of physiological signals employed as biofeedback depends on the therapeutic/target function of aVNS. For instance, peripheral blood flow can be used as a biofeedback signal when targeting peripheral vasodilation with aVNS, the tonus of the muscle excitation when targeting involuntary muscle contractions in dystonia, or heart rate and heart rate variability (HRV) from electrocardiogram when detecting stressful pain attacks or even suppressing atrial fibrillation via aVNS activated only once fibrillation is detected or predicted (Boon et al., 2016) using linear and/or non-linear methods (Pierzchalski et al., 2011; Nayak et al., 2018).

Instantaneous changes of the heart rate can be viewed as a phasic expression of central integrative processes in response to different tonic sensory signals. The frequency of heart rate changes is subjected to different competing mechanisms, which range from selective heart frequency entrainment to a specific physiological process, e.g., to the respiration cycle in terms of the respiratory sinus arrhythmia (Kaniusas, 2012), to changes in the gain of the feedback pathway (Fallen et al., 2001). For instance, VNS in a dog model was switched on when the heart interval dropped below a certain threshold in order to counteract arrhythmias which are favored by short heart intervals (Bilgutay et al., 1968). A closed-loop control of the heart rate was employed in pigs based on the invasive VNS (Tosato et al., 2006) where the stimulation frequency was controlled in order to select the right fiber type to be stimulated.

Not only cardiac-gated aVNS can be realized but also respiratory-gated aVNS (Kaniusas et al., 2009; Napadow et al., 2012). The temporal stimulation sequence of aVNS can be even synchronized with and follow to inner biological rhythms of the body to increase the coherence between residual body activities and aVNS effects (Figure 5). For instance, the relevance of the proper timing of VNS was shown for the induced ischemia and the following reperfusion in a swine model, with favorable VNS effects when applied before the reperfusion but not afterward (Shinlapawittayatorn et al., 2014). In general, VNS should be applied early in the course of the disease event or process (Stavrakis and Po, 2015).

Proper selection and targeted processing of physiological signals as recorded by the sensor (Figure 5) is of crucial importance for the closed-loop aVNS since the feedback should contain information about features of physiological reactions in response to aVNS. Here local biosignals can be used as the feedback within local control loops, e.g., monitoring the nerve excitation proximal to the nerve stimulation site (Ward et al., 2014), as well as global biosignals can input data into global control loops, e.g., monitoring of heart rate as a global outcome parameter (Ravan et al., 2017). Local loops can also be used to optimize the spatial and temporal distribution of the local stimuli based on personalized ear models while using distributed multiple needle electrodes in the ear. In contrast to local loops, global loops cover systemic physiological mechanisms (e.g., cardiorespiratory and cardiovascular) and show multi-scale dynamics (Siauciunaite et al., 2018) reflecting various body rhythms. Multimodal sensing – i.e., simultaneous usage of different sensing modalities – and/or multiparametric sensing – i.e., several parameters extracted from a single sensor – require minimum resources and deliver maximum information content, and thus can be expected to become powerful tools in the close-loop aVNS in future.

Figure 5A illustrates the closed-loop aVNS with the plethysmographic biofeedback, serving not only the controller to adjust the stimulus pattern but also synchronizing the stimulus. The target point defines the state to be achieved, e.g., the required blood perfusion level in the periphery or the targeted level of HRV. Since the individual human body as the system to be controlled is never sufficiently known and is subjected to continuous changes over time, adaptive methods (e.g., machine learning) should be used to define the controller. Figure 5B shows a simplified workflow of the closed-loop aVNS with the heart acting as the system to be controlled. Figure 5C shows a combination of Figure 5B with an individual ear model (from Figure 4B) derived from the transilluminated ear (from Figure 2D) in order to find personalized *and* optimized setting of stimulation needles and their stimulation patterns. Here the closed-loop aVNS re-optimizes continuously the stimulation patterns of respective electrodes to reach therapy-relevant targets.

### Therapeutic Applications

An individualized on-demand invasive VNS (controlled by patients with an active magnet stick) for epileptic treatment was a benefit for about 50% of patients as related to about 40% with the non-adaptive VNS (automatic intermittent VNS with inactive magnet) (Morris, 2003). Here the clinical benefit was defined in terms of aborted or decreased severity of seizures. In addition, the seizure improvement was unrelated to seizure frequency, whereas the on-demand approach provided a favorable reversal of “learned helplessness” to patients gaining a greater sense of control over their seizures.

The closed-loop VNS seems to be in favor of the open-loop VNS while reducing seizure severity (Boon et al., 2015). Here an increase of the heart rate of at least 20% – indicating an increased sympathetic outflow associated with ictal discharges (Eggleston et al., 2014) – was used to detect seizure onset and then to re-start VNS. Timely and personalized delivery of VNS in Ravan et al. (2017) was based on increased heart rate and synchronized brain dynamics to detect seizures for VNS therapy. The monitored heart rate can be used to close the VNS loop also in heart failure patients (Guiraud et al., 2016). In particular, the stimulation frequency, amplitude, pulse width as well as the on-off time of VNS – either synchronously or asynchronously with the R-wave of electrocardiogram – can be adjusted in response to the heart rate (Guiraud et al., 2016). The heart rate was also used as feedback for VNS affecting the atrioventricular node in order to reduce the heart rate in atrial fibrillation in a dog model (Zhang et al., 2002).

Auricular vagus nerve stimulation gated to the exhalation phase of respiration is suggested to be more efficient in the activation of NTS (Sclocco et al., 2019) and to improve analgesic benefits of aVNS while counteracting neuronal adaptation mechanisms (Napadow et al., 2012), especially in migraine patients (Garcia et al., 2017). The rationale of this approach is that natural activity of the efferent and afferent VN is tuned with respiration. In particular, VN activity is not only directly modulated by respiration via afferent VN endings in the lungs but also indirectly modulated via respiratory-related blood pressure changes (Kaniusas, 2012), namely, via the respiratory-related recruitment of VN afferents of baroreceptors (Karemaker, 2017).

## Future Directions

Auricular vagus nerve stimulation is a promising bioelectronic technology which may serve as an alternative, non-pharmacological, and neuro-immunomodulatory intervention. aVNS shows a variety of potential therapeutic applications due to its systemic effects on the human body (Kaniusas et al., 2019). Its huge potential, for instance, in chronic pain, warrants manifold research-guided and clinically related aVNS directions in future.

Founded investigations on the dose-response relationships in aVNS are sparse (Nonis et al., 2017) regarding relevant indications (Kaniusas et al., 2019), especially as a function of stimulation patterns and their parameters that would form a sound basis for the optimization of aVNS. Research-guided objectification and optimisation, as well as clinical validation of stimulation parameters are necessary based on functional PET/MRI/EEG with improved spatial and time resolution, while departing from purely empirical aVNS settings used currently for therapy in humans. In particular, parameters as intensity, duration, and timing of aVNS (i.e., neuromodulation protocols) could be optimized in *in silico* models, and then clinically validated in humans. The relevance and potential of a co-stimulation of non-vagal nerve endings in the auricle should be investigated further, revealing additional mechanisms of action and potential clinical applications of the electrical auricular stimulation. Possible synergetic effects of the afferent vagal and non-vagal stimulation in the cranial and spinal regions should be examined. Further research is needed to draw conclusions on efficacy and safety.

Individual optimization of stimulation patterns and their timing with respect to inner body rhythms – as based on the closed-loop aVNS – is another fundamental research line. A reasonable core set of sensor signals and their derived parameters should be established that best portray aVNS effects, as well as control algorithms permitting individualization at run time. Integrated sensors to track therapeutic progress and to control stimulation parameters are needed. Devices for aVNS should be integrated in telehealth solutions for a comprehensive closed-loop therapy management. Therapeutic benefit of the closed-loop aVNS has to be validated in relation to the open-loop aVNS.

Potential pairing of aVNS with other rehabilitative stimuli (Hays, 2015) – e.g., with rehabilitative limb movements in stroke (Dawson et al., 2015), with acoustical tones in tinnitus (De Ridder et al., 2013), or with extinction training (Peña et al., 2012) – should be investigated as promising option to increase efficacy of aVNS. This pairing is suggested to create targeted and correct maladaptive plasticity in the brain (Lehtimaki et al., 2012).

Easy-to-assess surrogate parameters and biomarkers should be derived to differentiate between aVNS responders and non-responders; e.g., VNS-induced release of norepinephrine was shown as a marker for seizure suppression (Raedt et al., 2011). Non-invasive markers of brainstem modulation by aVNS may include HRV as a potential candidate; e.g., stronger variability and higher vagal tone were observed in the responder group to VNS treatment (Liu et al., 2017), as well as event related potentials (De Taeye et al., 2014), pupillary diameter (Jodoin et al., 2015), and others (Mertens et al., 2018). Objective surrogate parameters could be even more useful to select responders than, for instance, subjective pain scores, especially for late responders to pain therapy.

## Conclusion

Auricular vagus nerve stimulation gains importance as a significant part of bioelectronics medicine in therapy, using easily controllable digital doses of electrical pulses instead of pharmaceutical drugs. Electroceuticals for aVNS provides promising means to modulate signaling between brain and periphery and thereby offers a window of opportunity for a disease-fighting effect on several disorders. aVNS is promising for systemic treatment, which can be easily interrupted, avoids severe side-effects, and is well-tolerated by patients. Currently, robust and validated stimulation protocols that are tuned to specific targeted physiological responses remain a great challenge in aVNS engineering. Beyond that a closed-loop aVNS needs to be established to account for individual physiological variability.

## Author Contributions

EK, SK, MT, and JS contributed to conception and design of the review. EK wrote the first draft of the manuscript. EK and SK performed the initial literature review. FP and MP contributed to anatomical sections of the manuscript. MT, RG, WK, and GV contributed to biological sections of the manuscript. SK and SL contributed to regulatory section of the manuscript. AC, ET, AS, TT, and WJ contributed to numerical section of the manuscript. VM and AL contributed to sections on personalized stimulation. NI and AŠ contributed to engineering sections on the manuscript. AK and BP contributed to clinical sections of the manuscript. All authors contributed to manuscript revision, read, and approved the submitted version.

## Conflict of Interest Statement

EK, SL, and SK were employed by SzeleSTIM GmbH. JS received honoraria from SzeleSTIM GmbH and owns patents in the field of the auricular vagus nerve stimulation. EK, SK, and JS were shareholders of SzeleSTIM GmbH. The remaining authors declare that the research was conducted in the absence of any commercial or financial relationships that could be construed as a potential conflict of interest.
